# The prevalence and risk of non-infectious comorbidities in HIV-infected and non-HIV infected men attending general practice in Australia

**DOI:** 10.1371/journal.pone.0223224

**Published:** 2019-10-09

**Authors:** Jack Edward Heron, Sarah M. Norman, Jeannie Yoo, Kirsty Lembke, Catherine C. O’Connor, Clare E. Weston, David M. Gracey

**Affiliations:** 1 Department of Renal Medicine, Royal Prince Alfred Hospital, Camperdown, New South Wales, Australia; 2 NPS MedicineWise, Sydney, New South Wales, Australia; 3 Kirby Institute, University of NSW, Sydney, New South Wales, Australia; 4 Central Clinical School, University of Sydney, Sydney, New South Wales, Australia; Institut Hospital del Mar d'Investigacions Mediques, SPAIN

## Abstract

**Background:**

Non-AIDS-related mortality rates among HIV-infected patients still exceed those of their uninfected peers. A major driver of this excess mortality is a higher risk of non-infectious comorbidities, including cardiovascular disease, chronic kidney disease, type 2 diabetes mellitus, osteoporosis and cancer. The prevalence of mental illness and other chronic non-infectious comorbidities is identified as a primary concern of antiretroviral prescribers in Australia.

**Methods:**

We conducted a cross-sectional, observational study using data from MedicineInsight, a large-scale Australian primary care database comprising longitudinal data from electronic clinical information systems. The HIV-infected cohort included all men with a recorded diagnosis of HIV. The non-HIV-infected cohort comprised all other men from the same practices. The prevalence and risk of cardiovascular disease, chronic kidney disease, type 2 diabetes mellitus, osteoporosis, cancer, anxiety and depression were compared between the groups.

**Results:**

We included 2,406 HIV-infected males and 648,205 males with no record of HIV diagnosis attending primary care in this study. HIV-infected men were less socioeconomically disadvantaged and more urban-dwelling than men in the primary care cohort. We found that HIV-infected men attending primary care in Australia are at increased risk of chronic kidney disease, cancer, osteoporosis, anxiety and depression. There appears to be a risk of premature onset of cardiovascular disease, osteoporosis and cancer among younger HIV-infected patients. There is a high prevalence of anxiety and depression among HIV-infected men.

**Conclusions:**

Increased prevalence of non-infectious comorbidities among HIV-infected men has broad implications for the effective management of those with these chronic conditions. Education to raise awareness among both HIV-infected men and their care providers, together with a greater focus on risk reduction, monitoring and preventive care, may be effective strategies in primary healthcare settings to further narrow the gap in health outcomes between people living with HIV and their uninfected counterparts.

## Introduction

The widespread use of highly active antiretroviral therapy and success in achieving durable viral suppression have led to dramatic demographic and epidemiological shifts in the population of people living with HIV. Non-AIDS-related causes of death now exceed AIDS-related illnesses as the major drivers of mortality among Australians living with HIV [1]. HIV-infected people are living longer and the surviving cohort is ageing [2]. Despite these advances, non-AIDS-related mortality rates among HIV-infected patients still exceed those of uninfected peers [1, 3].

HIV-infected patients are at an increased risk of a number non-infectious comorbidities (NICMs) including cardiovascular disease (CVD) [4], chronic kidney disease (CKD) [5], type 2 diabetes mellitus (T2DM) [6], osteoporosis [7], and cancer [8]. There is also increasing recognition of the high risk of mental illness among HIV-infected people, in particular anxiety and depression [9–11]. The management of NICMs is identified as one of the key clinical concerns of medical practitioners caring for HIV-infected people in Australia and the increasing burden of NICMs is a key driver for switching HIV treatments [12]. Mental health services and psychological support are identified as key unmet needs in the management of HIV-infected people [12].

The specific mechanisms underlying the difference in prevalence and risk of NICMs between HIV-infected and uninfected cohorts are not clear; HIV infection itself, antiretroviral toxicity, chronic immune activation and patient factors such as lifestyle and socio-economic status, among others, are likely to play a role. It remains unresolved whether NICMs occur at a younger age in HIV-infected people or whether there is a higher burden of NICM in HIV-infected people overall without a premature onset [13, 14].

There are no published population-based estimates of the prevalence of NICMs among HIV-infected people in Australia. We undertook this study to assess the prevalence of common NICMs in HIV-infected people in Australia; an area identified as clinically important but about which little has been published. Improving our understanding of NICM prevalence in HIV-infected people represents an opportunity to improve clinical outcomes with effective screening of at-risk patients and early modification of risk factors [15].

MedicineInsight is a national primary care data program developed and managed by NPS MedicineWise and is the first large-scale national primary care data collection program in Australia. MedicineInsight extracts longitudinal de-identified clinical data from primary care clinical information systems, and contains data on patient demographics, encounters, diagnoses, prescriptions, pathology tests and referrals. MedicineInsight includes prospectively gathered records for more than 3.5 million patients, with over 3,500 GPs from over 640 participating primary care practices. The patient population registered on the database is largely representative of the age, sex and geographical distribution of the Australian population [16].

We have used data from MedicineInsight to investigate the prevalence and risk of seven common NICMs (depression, anxiety, cancer, CVD, osteoporosis, T2DM and CKD) among HIV-infected men compared to men without HIV who attend Australian general practices enrolled in MedicineInsight. We restricted the results and discussion presented here to male patients only, to eliminate sex as a confounder between the two groups.

## Methods

### Data collection

A cross-sectional observational study was conducted using the June 2017 MedicineInsight download. Data were extracted from all relevant available data fields including patient demographics, diagnoses and symptoms, observations and referrals. Progress notes containing free text are not available, as they can incorporate patient-identifying information. Patient location was assigned using the Australian Bureau of Statistics (ABS) mapping of the patient’s postcode of residence to Australian Statistical Geography Standard (ASGS) Remoteness Areas 2011 [17]. Socio-Economic Indexes for Areas (SEIFA) were assigned based on patient postcode and calculated in accordance with the ABS Index of Relative Socio-Economic Advantage and Disadvantage (IRSAD) quintiles [18].

### Study population

Patients were selected for inclusion in the study if they were aged 18 years or over, alive, marked as active by the practice, had 3 or more visits to the practice in the past 2 years, and were treated in a primary care practice that met the following MedicineInsight standard data quality requirements:

- established as a practice for at least 2 years, to ensure adequate longitudinal data on patients
- no gaps of more than 2 months in the previous 2 years in data entry into key data tables (patients, diagnoses or patient history, encounters, observations, prescriptions, pathology test requests and results), and
- data available for at least 50 patients in the 2 years prior to the database build, to exclude practices that did not record clinical data in their CISs.

The cohort of HIV-infected patients was defined as those with a recorded diagnosis of HIV at any time in their medical history, in any designated text or code field in the diagnosis tables, reason for prescription, or reason for visit. Patients were excluded if their HIV had been diagnosed within the preceding 6 months, as we were specifically looking at comorbidities associated with longer term HIV infection.

The general primary care population cohort was drawn from all other eligible active patients attending the same general practices from which the HIV-infected patient cohort was selected, and for whom there was no indication of a diagnosis of HIV or AIDS.

### Clinical definitions

We have investigated the recording and relative risk of specific NICMs in HIV-infected people and the general practice population. We developed coding algorithms to identify conditions and symptoms of interest within the MedicineInsight database, using commonly accepted clinical definitions, terms and synonyms from SNOMED CT-AU [19]. Clinical conditions were derived from medical classification system (DOCLE and Pyefinch)-coded and free-text data fields including diagnosis, medical history, reason for encounter (ie, reason for visit or consultation) and reason for prescription.

For NICMs, the data were coded based on the dichotomous outcomes of “ever been diagnosed within lifetime” and “no record of diagnosis”. While this is not intended to be representative of the overall lifetime prevalence of the condition, the recorded rates for CVD and T2DM in the MedicineInsight dataset are similar to reported national prevalence rates [16].

### Statistical analysis

Descriptive statistics, including percentages, relative risks and associated 95% confidence intervals (CI), have been used to describe the cohort of HIV-infected patients and the general population comparator according to socio-demographic characteristics and risk factor profile. The significance of the effect of age, rurality or socioeconomic status (as indicated by SEIFA quintiles) was assessed using multivariable analysis and determination of odds ratios (ORs) with associated 95% CIs, based on the final logistic model fitting. Analyses were conducted using SPSS Statistics v23 (IBM).

### Limitations of the study

General practice data collection serves the primary purpose of managing patient care. The quality and completeness of MedicineInsight data are dependent on the accuracy and completeness of data recorded in, and available from, the general practice clinical information systems.

A limitation of MedicineInsight is that patients are not linked across practices, and patients who regularly visit more than one practice that contributes to MedicineInsight may be recorded more than once. It has been estimated that approximately 4% of general population patients may be duplicates [16].

### Ethical approval

This study was approved by the Bellberry Human Research Ethics Committee (2016-10-781).

## Results

We identified 2,630 HIV-infected patients and 1,522,550 general population patients who met the study population selection criteria. The HIV-infected cohort was 91.5% male, with 2,406 males and 222 females. In comparison, the general practice population was 57.3% female and 42.6% male (872,662 and 648,205 patients respectively). We therefore restricted the results presented here to male patients only, in both HIV-infected and uninfected cohorts, to eliminate sex as a confounder, ensure adequate statistical power (particularly upon stratification by age) and to reduce the potential risk of patient re-identification due to small cell sizes (Table 1).

**Table 1:** Patient socio-demographic characteristics.

Characteristics	Male HIV-infected (N = 2,406)		Male general practice population (N = 648,205)	
	Number	%	Number	%
Age group				
18–29 years	95	3.9	107,170	16.5
30–44 years	591	24.6	159,760	24.6
45–64 years	1,417	58.9	216,630	33.4
65–74	248	10.3	94,697	14.6
75+	55	2.3	69,948	10.8
Patient rurality				
Major cities	1,925	80.0	407,328	62.8
Inner regional	356	14.8	156,787	24.2
Outer regional, remote and very remote	108	4.5	79,884	12.3
Missing	17	0.7	4,206	0.6
Patient SEIFA^a^ quintile				
1 –more disadvantaged	213	8.9	112,833	17.4
2	274	11.3	102,797	15.9
3	400	16.6	138,167	21.3
4	441	18.3	123,256	19.0
5 –least disadvantaged	1,059	44.0	165,853	25.6
Missing	19	0.8	5,299	0.8

^a^ Socio-Economic Indexes for Areas (SEIFA) is a product developed by the Australian Bureau of Statistics that ranks areas in Australia according to relative socio-economic advantage and disadvantage. The indexes are based on information from the 5-yearly Census.

The demographic distribution of the HIV-infected cohort, as predominantly urban-dwelling men aged 45–64 years, differs significantly from that of the general practice population, consistent with previously reported HIV demographic and prevalence characteristics in Australia [2]. Almost 94% of HIV-infected males were aged between 30 and 74 years, and there was a significantly greater proportion of HIV-infected males aged between 45 and 64 years (Table 1). Compared to the male general practice population, there were fewer HIV-infected males aged under 30 years (3.9% vs 16.5%) or aged 75 and above (2.3% vs 10.8%). There was also a higher proportion of male HIV-infected patients in the least disadvantaged SEIFA quintile areas (44%) compared to the male general practice population (25.6%).

**Fig 1:**
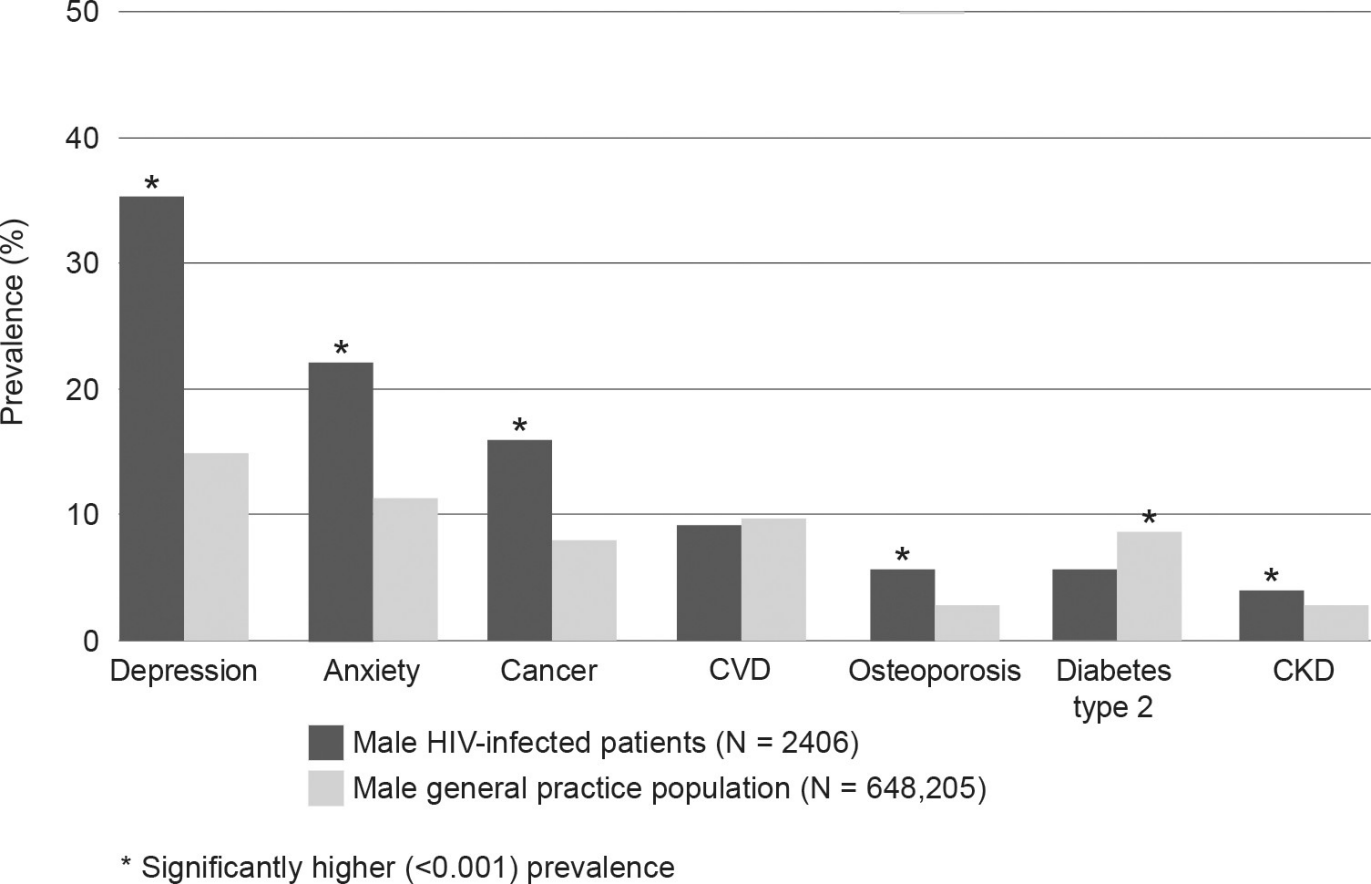
Overall prevalence (%) of NICMs in male HIV-infected patients compared to the male general practice population. *p < 0.001.

Male HIV-infected patients had statistically significantly higher (p < 0.001) overall prevalence rates of depression, anxiety, cancer, osteoporosis and CKD compared to the male general practice population (Fig 1). There was no significant difference in the overall prevalence of CVD demonstrated between the two groups. T2DM was significantly more prevalent in the general practice population.

We investigated the age-stratified odds ratio (OR) of these NICMs, adjusting for rurality and socioeconomic status (SEIFA quintile), and additionally adjusting for age in the overall prevalence analysis (Table 2). We found that the prevalence and risk of both depression and anxiety were significantly higher in HIV-infected males in all age groups except those aged over 75 years, compared to the male general practice population. The overall prevalence of depression and anxiety in the male HIV-infected group was 35.5% and 22.9% compared to 15.1% and 10.9% respectively in the male general practice population, and the overall risk of depression and anxiety was more than doubled in the HIV-infected group (OR 3.0, 95% CI 2.8 to 3.4 and OR 2.4, 95% CI 2.2 to 2.6 respectively).

**Table 2:** Proportion and adjusted odds ratio of selected NICMs in male patients by HIV status and age group.

Proportion (%) of patients with depression				
Age group (years)	Male HIV-infected	Male general practice population	Adjusted odds ratio* (95% CI)	P value
18–29	25.3%	13.0%	2.391 (1.501, 3.809)	.000
30–44	36.0%	15.5%	3.527 (2.976, 4.181)	.000
45–64	38.0%	17.0%	3.167 (2.840, 3.531)	.000
65–74	28.2%	14.1%	2.477 (1.873, 3.274)	.000
75+	16.4%	13.1%	1.427 (0.696, 2.925)	.332
TOTAL	35.5%	15.1%	**3.008 (2.837, 3.361)	.000
Proportion (%) of patients with anxiety				
Age group (years)	Male HIV-infected	Male general practice population	Adjusted odds ratio* (95% CI)	P value
18–29	24.2%	12.3%	2.291 (1.430, 3.668)	.001
30–44	29.3%	12.9%	2.836 (2.371, 3.392)	.000
45–64	22.5%	10.9%	2.333 (2.055, 2.647)	.000
65–74	13.7%	8.2%	1.777 (1.236, 2.554)	.002
75+	5.5%	7.7%	0.726 (0.226, 2.328)	.590
TOTAL	22.9%	10.9%	**2.376 (2.158, 2.616)	.000
Proportion (%) of patients with cancer				
Age group (years)	Male HIV-infected	Male general practice population	Adjusted odds ratio* (95% CI)	P value
18–29	2.1%	0.5%	4.069 (0.999, 16.570)	.050
30–44	3.7%	1.2%	3.257 (2.120, 5.002)	.000
45–64	15.0%	6.5%	2.561 (2.208, 2.969)	.000
65–74	29.8%	18.3%	1.914 (1.456, 2.514)	.000
75+	34.5%	28.2%	1.344 (0.760, 2.375)	.309
TOTAL	13.7%	8.3%	**2.363 (2.089, 2.672)	.000
Proportion (%) of patients with cardiovascular disease (CVD)				
Age group (years)	Male HIV-infected	Male general practice population	Adjusted odds ratio* (95% CI)	P value
18–29	1.1%	0.1%	9.560 (1.319, 69.260)	.025
30–44	0.8%	0.7%	1.415 (0.585, 3.423)	.441
45–64	8.9%	6.8%	1.399 (1.162, 1.684)	.000
65–74	29.0%	20.9%	1.595 (1.211, 2.101)	.001
75+	41.8%	38.2%	1.086 (0.626, 1.885)	.769
TOTAL	9.4%	9.6%	**1.405 (1.215, 1.625)	.000
Proportion (%) of patients with osteoporosis				
Age group (years)	Male HIV-infected	Male general practice population	Adjusted odds ratio* (95% CI)	P value
18–29	0.0%	0.1%	NA	NA
30–44	1.0%	0.2%	6.758 (2.988, 15.286)	.000
45–64	6.2%	0.9%	7.718 (5.753, 8.956)	.000
65–74	12.1%	3.5%	3.797 (2.587, 5.574)	.000
75+	10.9%	11.4%	0.965 (0.412, 2.259)	.934
TOTAL	5.4%	2.1%	**5.058 (4.196, 6.097)	.000
Proportion (%) of patients with type 2 diabetes mellitus (T2DM)				
Age group (years)	Male HIV-infected	Male general practice population	Adjusted odds ratio* (95% CI)	P value
18–29	0.0%	0.2%	NA	NA
30–44	1.0%	1.8%	0.625 (0.279, 1.398)	.253
45–64	6.7%	9.0%	0.767 (0.621, 0.946)	.013
65–74	11.3%	18.1%	0.597 (0.403, 0.886)	.010
75+	12.7%	21.1%	0.493 (0.211, 1.155)	.103
TOTAL	5.7%	8.4%	**0.697 (0.584, 0.832)	.000
Proportion (%) of patients with chronic kidney disease (CKD)				
Age group (years)	Male HIV-infected	Male general practice population	Adjusted odds ratio* (95% CI)	P value
18–29	0.0%	0.1%	NA	NA
30–44	0.5%	0.2%	2.429 (0.776, 7.600)	.127
45–64	2.6%	0.8%	3.556 (2.554, 4.951)	.000
65–74	8.1%	2.8%	3.263 (2.060, 5.170)	.000
75+	9.1%	7.7%	1.056 (0.380, 2.930)	.917
TOTAL	2.7%	1.6%	**3.006 (2.329, 3.878)	.000

*adjusted for SEIFA and rurality

**adjusted for age, SEIFA and rurality

Overall prevalence and risk of all-cause cancers were significantly higher for male HIV-infected patients. HIV-infected males aged 30–44 were three times more likely to have had a diagnosis of cancer than the age-matched patients from the general practice population (OR 3.3, 95% CI 2.1 to 5.0). Cancer rates were also significantly higher in HIV-infected males aged between 45 and 74 years. Although not statistically significant, there was also a higher risk of cancer in HIV-infected patients aged 18–29 years (OR 4.1, 95% CI 1.0 to 16.6). Cancer prevalence increased with age in both HIV-infected and general practice population male patients.

There was a small but significant difference in overall CVD prevalence between male HIV-infected and male general practice population patients (OR 1.4, 95% CI 1.2 to 1.6). When stratified by age, there was a significantly higher risk of CVD in the younger male HIV-infected patients aged 18–29 years and in male HIV-infected patients aged from 45–64 years and 65–74 years.

The overall risk of osteoporosis was higher in male HIV-infected patients compared to the male general practice population (OR 5.1, 95% CI 4.2 to 6.1). Rates of osteoporosis were also significantly higher in male HIV-infected patients aged 30–74 years, and HIV-infected patients aged 30–44 and 45–64 years were at least six times more likely to have been diagnosed with osteoporosis than males in the general practice population (OR 6.8 and 7.7, 95% CIs 3.0 to 15.3 and 5.8 to 9.0 respectively).

T2DM was the only NICM investigated for which the overall risk was significantly lower in the HIV-infected males compared to the male general practice population (OR 0.7, 95% CI 0.6 to 0.8). While T2DM was relatively uncommon in younger male patients from either the HIV-infected or general practice population groups, prevalence and risk increased with age, and risk was significantly lower in the HIV-infected population aged 45–64 and 65–75 years compared to the general practice population (OR 0.8, 95% CI 0.6 to 0.9 and OR 0.6, 96% CI 0.4 to 0.9 respectively). CKD diagnosis rates were also low in younger age groups, but were significantly higher in HIV-infected males in the 45–64- and 65–74-year age groups (OR 3.6, 95% CI 2.3 to 4.4 and 3.3, 95% CI 2.1 to 5.2).

**Fig 2:**
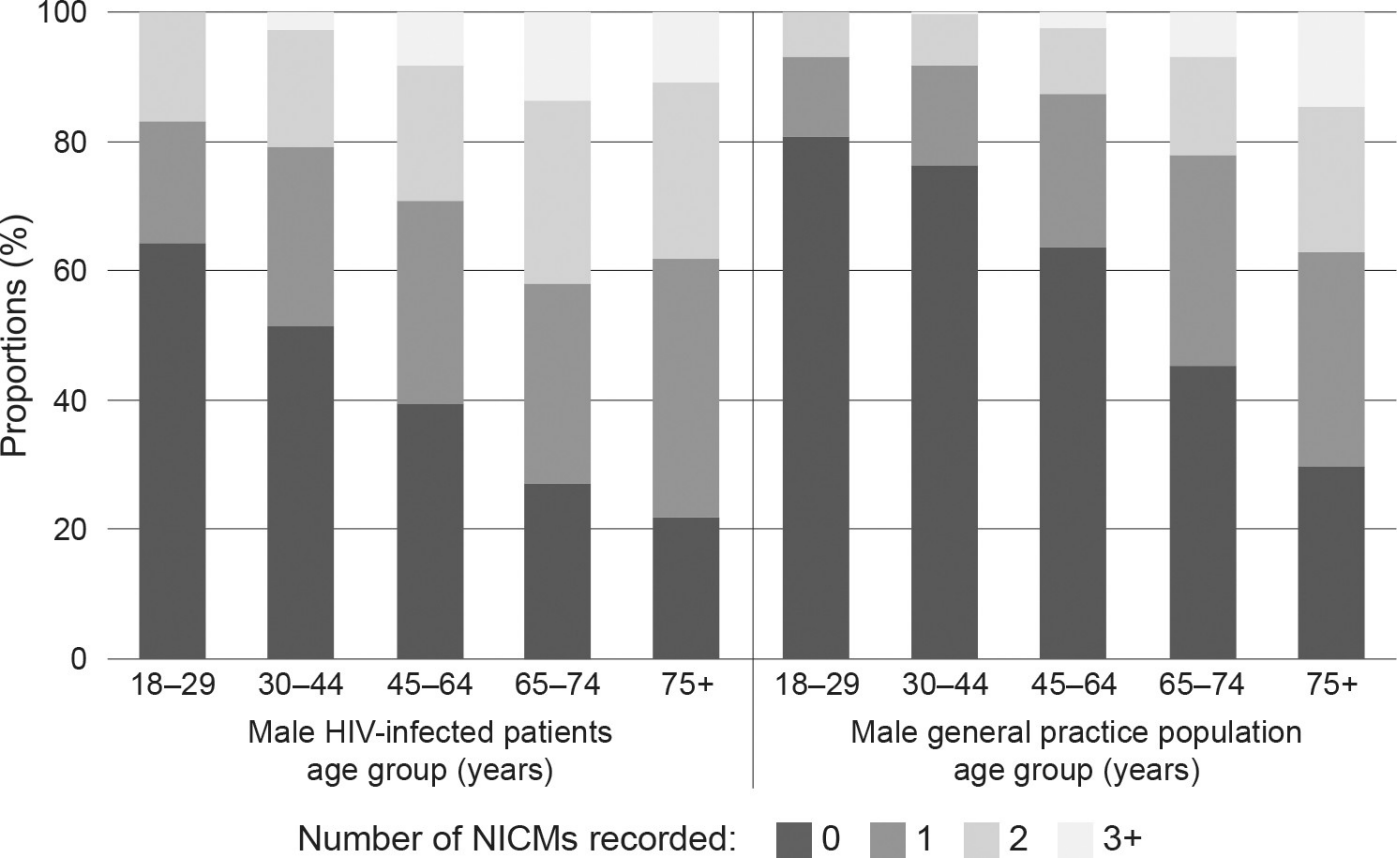
Multiple NICMs recorded by HIV status and age group.

Our results also show that 28% of HIV-infected males had 2 or more of the NICMs investigated here compared with only 17% of the male general practice population, while only 41.6% of male HIV-infected patients had no NICMs, compared with 63.2% of the male general practice population (Table 3). The risk of multiple comorbidities was significantly higher in younger HIV-infected males; however, the trend towards more patients with multiple comorbidities in HIV-infected males was apparent across all age groups examined in this study (Fig 2).

**Table 3:** Multiple NICMs recorded in male HIV-infected patients compared to the male general practice population.

Number of comorbid conditions recorded	Male HIV-infected		Male general practice population	
	Number	%	Number	%
0	1002	41.6	409489	63.2
1	726	30.2	143596	22.2
2	505	21.0	71892	11.1
3	137	5.7	17874	2.8
4 or more	36	1.5	5354	0.8

## Discussion

In this study of patients attending primary care practices in Australia we found a higher prevalence of depression, anxiety, cancer, osteoporosis and CKD in HIV-infected males compared with the male general practice population. We found a lower prevalence of T2DM in the HIV-infected cohort. While the overall prevalence of CVD was no different between the groups, after adjusting for age, socioeconomic status and rurality HIV-infected males had a significantly higher risk of CVD than the general practice population. Our findings confirm a higher burden of NICM among HIV-infected men in Australia.

Data on the prevalence and risk of depression among people living with HIV are restricted to small methodologically limited studies that estimate the point-prevalence of depression to be between 20% and 30% [10, 20]. In an older cohort, HIV infection was not found to be associated with an increased rate of anxiety symptoms or disorders [21]. In our cohort, HIV-infected men had a higher overall prevalence of depression of 35.5% and were at a consistently increased risk of having been diagnosed with depression across all age groups up to 75 years. A similar pattern of risk was seen for anxiety with an overall prevalence of 22.9%. These relationships were unchanged after adjustment for socioeconomic status and rurality. Our data are limited by the quality of diagnostic coding in general practice clinical information systems which are intended primarily for patient care. These estimates likely include patients with presentations not meeting diagnostic criteria for specific anxiety disorders or depressive disorder in both the HIV-infected and non-infected groups. Nonetheless they provide a clinically meaningful estimate of the burden of mental illness among people living with HIV and provide important context for Australian antiretroviral prescribers who rank mental illness and psychological support as unmet service needs. In addition to the associated morbidity and mortality, patients with comorbid mental illness are less likely to achieve viral suppression when commencing antiretroviral therapy [22] which further emphasises the implications of these findings for individual patients and public health programs more broadly. Anxiety and depression represent an under-recognised epidemic among HIV-infected men in Australia, and our findings indicate the clear need for further research and clinical action.

The antiretroviral era has seen a fall in the incidence of AIDS-defining cancers like Kaposi sarcoma and non-Hodgkin lymphoma [23]. While the incidence of non-AIDS-defining cancers now exceeds that of AIDS-defining cancers, the effect of modern antiretroviral therapy and HIV infection itself on the incidence of non-AIDS-defining cancers remains unclear. Large series have reported either no difference in incidence [23] or a significantly increased standardised incidence ratio of up to 2.5 among HIV-infected patients [8]. Some series suggest a premature onset of non-AIDS-defining cancer risk with no increased relative risk among older HIV-infected patients [14]. We report a significantly higher prevalence of cancer among HIV-infected men compared with other men attending primary care (13.7% versus 8.3%), with a consistently increased risk of having been diagnosed with a cancer among HIV-infected men between the ages of 30 and 75 years. These relationships are unchanged after adjusting for socioeconomic status and rurality. We observed the highest relative risk in the youngest patients, reflecting a small increase in the absolute risk of an age-related disease. A similar pattern of increased relative risk in younger patients was seen in the risk of osteoporosis and cardiovascular disease in our cohort; two other age-related diseases. This excess risk may be attributable to virus-associated cancers, in particular among HIV-infected patients who smoke tobacco [24]. A limitation of our data is an inability to distinguish between AIDS-defining cancers, non-melanoma skin cancers and solid organ cancers. Nonetheless our data suggests an increased and premature risk of cancer among HIV-infected men and supports further research focused on cancer screening and prevention in this population.

HIV-infected patients have a 1.5 to 2.5-fold increased risk for major cardiovascular events [4]. We report a similar prevalence of cardiovascular disease among HIV-infected men and non-HIV-infected men attending primary care in Australia (9.4% versus 9.6%). The HIV-negative cohort however were on average older than the those with HIV. After adjusting for age, socioeconomic status and rurality, HIV-infected men had a significantly increased risk of CVD, which is consistent with other published estimates. The relative risk of CVD among HIV-infected men aged 45–64 years was significantly increased, at around 1.4 times higher than for non-HIV-infected men. A similar pattern of increased risk in younger HIV-infected patients has been reported in other series [25, 26].

The association between HIV infection and osteoporosis is well described [27]. Antiretroviral therapy, in particular tenofovir disoproxil fumerate, is associated with bone demineralisation at both the hip and spine [7]. Other risk factors for osteoporosis, including alcohol misuse, tobacco use and low body mass index (BMI) may be more common among Australian HIV-infected patients [27]. We report an overall prevalence of osteoporosis of 5.4% with a pattern of risk similar to cardiovascular disease and cancer; highest in younger age groups and tapering to parity with those aged 75 years and older. The prevalence of osteoporosis among HIV-infected men aged 45–74 years ranged from 6.2% to 12.1%. Comparisons between published series are confounded by different inclusion criteria and case definitions. Osteoporosis was not reported in the APPLES study examining self-reported comorbidities among older HIV-infected Australian men [6] and the prevalence we report is lower than in a prospective cohort of patients aged 45 years and older recruited from Dutch HIV clinics [28]. It seems unlikely that patients would present complaining of “osteoporosis” in the way they might present with “anxiety” or “depression” not necessarily meeting diagnostic criteria. Given clear radiographic and clinical definitions exist, we believe our data reflect rates of diagnosed osteoporosis in Australia.

It is well established that HIV-infected patients are at increased risk of chronic kidney disease [14, 28]. In an Australian series, 4.2% of HIV-infected patients developed CKD in an 8-year follow-up period [5]. CKD is known to be under-recognised and underdiagnosed in Australia [29]. We report a prevalence of diagnosed CKD of 2.7% among HIV-infected men in Australia compared with 1.6% in the general practice population. This is the first reported estimate of prevalence of diagnosed CKD among HIV-infected men in Australia. Our estimate of the prevalence of diagnosed CKD in the general practice cohort is consistent with other Australian estimates [29]. Our estimate of diagnosed CKD prevalence among HIV-infected patients is lower than international series [14, 28, 30]. This may reflect underdiagnosis, a younger cohort, local ART prescribing patterns, and variation in the prevalence of comorbid risk factors for kidney disease including hepatitis virus infection, APOL1 polymorphisms, hypertension and diabetes mellitus. We found the risk of CKD was highest among HIV-infected men between the ages of 45 and 74 years (OR 3.3 to 3.6). Among older HIV-infected men we found CKD prevalence approximated that in the general practice population. Unlike CVD, there was no trend towards a marked increase in risk in younger HIV-infected men. The prevalence of CKD has increased in the antiretroviral era and while multifactorial, this is likely most attributable to drug toxicity and comorbid risk factors for CKD progression [31, 32]. HIV-infected patients with CKD and albuminuria are at increased risk of cardiovascular disease and prevalent CKD may be one factor driving CVD risk in this population [33].

An association between HIV infection and risk of T2DM has not been confirmed. Published series report either an increase in risk or no difference in risk among HIV-infected subjects [6, 14, 28]. We report a significantly lower prevalence of T2DM in our HIV cohort compared with the general practice cohort (5.7% vs. 8.4%). In the APPLES study, HIV-infected men were more obese but exercised the same amount as non-HIV-infected peers. The APPLES study reported a significantly higher prevalence of self-reported diabetes mellitus in the HIV-infected cohort of approximately 15% [6]. Australian HIV-infected patients can attend public hospitals and free sexual health clinics for their HIV care. MedicineInsight does not include data from these providers, which may disproportionately serve more disadvantaged patients. Our finding of a lower prevalence of T2DM is striking and could also be explained by bias in sampling methods or disproportionate underdiagnosis of T2DM in the general practice cohort. The overall prevalence of T2DM in the MedicineInsight dataset accurately approximates that of the general population which strongly supports the robustness of our estimate [16].

In conclusion, the increasing prevalence of NICMs among people living with HIV has led to increased complexity of care and has implications for the screening, diagnosis and effective management of these chronic conditions in healthcare settings more accustomed to the management of acute HIV-related presentations. Raising awareness of the increased prevalence of NICMs among HIV-infected men and their care providers and placing a greater focus on reduction of risk and preventive care in the primary care setting may be effective strategies to further narrow the gap in health outcomes between people living with HIV and their non-infected counterparts.
